# Prevalence and correlates of intimate partner violence among women with HIV in serodifferent relationships in Nairobi, Kenya

**DOI:** 10.1371/journal.pone.0272640

**Published:** 2022-08-17

**Authors:** Anne Kaggiah, Katherine Wilson, Barbra A. Richardson, John Kinuthia, Carey Farquhar, R. Scott McClelland

**Affiliations:** 1 Research and Programs Department, Kenyatta National Hospital, Nairobi, Kenya; 2 Department of Global Health, University of Washington, Seattle, Washington, United States of America; 3 Department of Biostatistics, University of Washington, Seattle, Washington, United States of America; 4 Department of Medicine, University of Washington, Seattle, Washington, United States of America; 5 Department Epidemiology, University of Washington, Seattle, Washington, United States of America; 6 Department of Medical Microbiology, University of Nairobi, Nairobi, Kenya; Flinders University, AUSTRALIA

## Abstract

**Background:**

Intimate partner violence (IPV) is a major public health problem and is the most common form of violence against women. Women with HIV in serodifferent relationships may be at an increased risk of IPV compared to women without HIV, hindering their ability to adhere to antiretroviral therapy, clinic appointments, and condom use during sex. This study assessed the prevalence and correlates of IPV in the past year among women with HIV in serodifferent relationships in Nairobi, Kenya.

**Methods:**

This cross-sectional study included women with HIV in serodifferent relationships who were at least 18 years old and provided written informed consent. Their experience of physical, sexual, or emotional violence in the past year by the current partner was assessed using 13 questions adapted from the World Health Organization survey on violence against women. Standardized instruments were used to assess sociodemographic and behavioral factors. Associations between intimate partner violence and other variables were evaluated using log binomial regression models.

**Results:**

Of the 159 women enrolled, 47 (29.6%, 95% CI 22.9–37.2%) reported IPV in the past year. Of these, 32 (68.1%) reported emotional, 27 (57.4%) physical, and 27 (57.4%) sexual violence. In the multivariate model, pregnancy (adjusted prevalence ratio [aPR] 2.14, 95% CI 1.09–4.20), alcohol use (minimal drinking aPR 1.91, 95% CI 1.10–3.33; moderate/severe drinking aPR 1.17, 95% CI 0.53–2.59), male partner controlling behavior (aPR 2.09, 95% CI 1.24–3.51), and past physical violence (aPR 1.93, 95% CI 1.22–3.05) remained significantly associated with a higher prevalence of IPV in the past year.

**Conclusion:**

This study identified a high prevalence of IPV in the past year among women with HIV in serodifferent relationships. Pregnant women and women who had experienced prior violence had a higher prevalence of IPV. These data highlight the need to screen for IPV during clinic visits, and to offer evidence based interventions to support women in serodifferent relationships who have experienced IPV.

## Introduction

Intimate partner violence (IPV) is defined as behavior within an intimate relationship that causes physical, sexual, or emotional harm, and is the most common form of violence against women (VAW) [1, 2]. It is estimated that 30% of women experience IPV in their lifetime, making it a global health concern [2, 3]. In Kenya, the 2014 Demographic and Health Survey showed that 41% of ever married women reported lifetime IPV, while 23% reported IPV in the past year [4].

According to the ecological framework of violence against women, a combination of individual, relationship, community, and societal factors play a role in IPV among women [5, 6]. Individual risk factors include age, pregnancy, alcohol, and a prior history of violence. The risk of IPV is generally higher in younger women [6, 7]. There are conflicting findings on whether pregnancy increases or reduces the risk of IPV [8, 9]. A review of studies in developed countries showed higher risk, while a review of African studies showed lower risk of IPV for women during pregnancy [10, 11]. There is evidence suggesting that alcohol use among women predisposes them to IPV [7, 12, 13]. Previous history of violence has also been consistently associated with higher risk of IPV [7, 14].

Relationship-level risk factors for IPV include marital status and male partner controlling behaviors [5, 7]. Some studies show that married women are at increased risk of IPV, while others show reduced risk compared to women who are single or divorced [7]. A literature review of studies conducted in the United States found that separated or divorced women were at higher risk of IPV compared to married women [7]. The reviewed data suggested that women may experience IPV during times of relationship stress, such as negotiations with a male partner about children or property [15]. Married women also experience IPV, particularly in the setting of ongoing marital conflict [6, 7]. Women who have male partners with controlling behaviors are at increased risk of IPV [12, 16].

Community-level risk factors for IPV include poverty and lack of family support [6, 17]. Women experiencing poverty may be financially dependent on their partners and lack alternative means of survival, making it difficult to leave abusive relationships. Additionally, those who lack family support are likely to be dependent on their male partners, and not report abuse [17]. At a societal level, violence against female partners is viewed as the norm in some African cultures, and women are expected to endure abuse from their partners without complaining or seeking help [17, 18]. Lack of legislation categorizing IPV as a criminal offence may also perpetuate IPV [6, 17].

Numerous studies have suggested that human immunodeficiency virus (HIV) infection predisposes women to IPV [12, 19, 20]. Women in serodifferent relationships may experience IPV after disclosure, where lack of trust and concerns regarding infidelity as a possible source of the HIV infection may lead to conflict [21, 22]. In addition, women with HIV in serodifferent partnerships may suffer from IPV-related outcomes such as depression, post-traumatic stress disorder, alcohol and substance use, all of which may interfere with their adherence to antiretroviral therapy (ART) and attendance of clinic appointments, hindering efforts to manage HIV and reduce transmission risk [23].

Improving our understanding of the correlates of IPV in women with HIV in serodifferent relationships can help to identify women at risk, inform interventions to reduce IPV, improve HIV-related health outcomes, and reduce the risk of transmission to partners and infants [12]. This study aimed to determine the prevalence and correlates of IPV in the past year among women with HIV in serodifferent relationships in Kenya.

## Methodology

### Study design

This was a cross-sectional analysis of baseline data from a cohort study of women with HIV in serodifferent relationships. The study was conducted at the Couples Counselling Center located in Kenyatta National Hospital (KNH), the largest referral and teaching hospital offering specialized health care services in Nairobi, Kenya. The Couples Counselling Center offers risk reduction education, ART, safer conception counseling, and screening and treatment for sexually transmitted infections (STIs) to serodifferent couples in and around Nairobi. Sample size calculation was not performed specifically for this secondary analysis, which used data from an existing study.

### Recruitment and enrollment procedures

Study staff visited the KNH Voluntary Counselling and Testing Centers (VCTs), the KNH Comprehensive Care Center, and other VCTs around Nairobi. During these visits, study staff informed health care providers about the study and requested referral of HIV-serodifferent couples.

The couples who presented at the Couples Counselling Center were tested for HIV using rapid tests according to national guidelines. Prior to testing, couples counseling was conducted and couples were encouraged to mutually disclose their HIV status. Female partners with HIV who were at least 18 years of age and provided written informed consent were eligible to participate. Seronegative male partners were encouraged to access free follow-up services offered to both members of the serodifferent couple at the clinic.

Ethical approval was obtained from the KNH/University of Nairobi Ethics and Research Committee (P42/02/2013) and the University of Washington Human Subjects Research Committee (Study00004960).

### Inclusivity in global research

Additional information regarding the ethical, cultural, and scientific considerations specific to inclusivity in global research is included in the S2 Checklist.

### Data collection

At enrollment, women completed a standardized face-to-face interview in their preferred language (Kiswahili or English), conducted in a private room by trained study staff. The interview included questions on socio-demographics, reproductive health, and sexual risk behaviors.

Interview questions about IPV were adapted from the World Health Organization (WHO) survey on VAW, an instrument that is standardized and has good internal consistency [24]. Participants were asked to focus on their current partners when responding to six questions about any past history of physical violence (slapped, kicked, hit, threatened with a weapon, choked, or pushed), four about emotional violence (insulted, intimidated, belittled, or threatened someone they cared about), and three about sexual violence (forced sex, coerced sex, forced to perform a degrading sexual act). Women who reported any lifetime history of IPV were then asked whether specific acts of violence occurred during the past year. Women were also asked about controlling behaviors of their current partner. These behaviors are recognized to be distinct from IPV [25], and included insisting on knowing the woman’s whereabouts at all times, restricting contact with her family or friends, and controlling her access to health care [25]. Women who responded ‘yes’ to at least one of the seven statements about controlling behaviors were considered exposed. Participants were also asked about violence since age 15 by someone other than their current partner. Participants who reported any IPV were counseled by the study staff and referred for care at KNH Gender Based Violence and Recovery Center as needed.

Alcohol use was assessed using the WHO alcohol use disorders identification test (AUDIT) with a score range of 0 to 40. Scores were grouped as 0 (no drinking), 1–6 (minimal drinking), 7–15 (moderate drinking), and ≥16 (severe drinking or possible alcohol use disorder) [26]. The last two categories were collapsed into a single category representing moderate/severe drinking because few participants had AUDIT scores ≥16. Screening for depression was conducted using the patient health questionnaire (PHQ-9) with a score range of 0 to 27, where scores were categorized as 0–4 (minimal), 5–9 (mild), ≥10 (major depressive disorder) [27, 28].

Following the interview, the study clinician conducted a physical examination including a speculum-assisted pelvic examination with collection of genital swabs for laboratory detection of STIs. Following the study visit, participants received 300 Kenyan shillings (approximately 3 US dollars) to compensate them for their transportation expenses.

### Laboratory procedures

Testing for HIV was performed using Determine HIV-1/2 (Inverness Medical, Waltham, MA), and confirmed using the HIV-1/HIV-2 3rd generation enzyme linked immunoassay (Abbott Molecular, Ontario). Pregnancy testing was performed on urine samples using a rapid test (QuickVue, Quidel, San Diego, CA).

### Data analyses

The main outcome was any IPV in the past 12 months. Women were considered to be exposed if they responded ‘yes’ to at least one of 13 questions about acts of IPV [27]. Potential correlates of IPV in the past year were selected for evaluation based on a review of literature. Socio-demographic characteristics included age [6, 7], years of education [5, 6], and marital status [6, 7]. Reproductive characteristics included pregnancy (confirmed by urine B-hCG) [5, 6], number of live births [6], fertility desire (wants more children) [29], fertility intent (currently trying to become pregnant) [21], and use of modern non-barrier contraception (oral contraceptive pills [OCP], implant, depot medroxyprogesterone acetate [DMPA], intrauterine device [IUD], tubal ligation, and hysterectomy) [30]. Partner’s attitude towards a possible pregnancy (excited, would not care, upset), and the number of sexual partners in the past week were also assessed [9, 31].

The prevalence of past year IPV was calculated with 95% confidence intervals (CIs). Correlation matrices and graphics (e.g. scatter plots and box plots) were used to check for collinearity. Univariate log binomial models were used to estimate the association between each correlate and past year IPV. Subsequently, variables associated with past year IPV in univariate analysis (p < 0.10) were included simultaneously in a multivariate model to identify variables that were independently associated with IPV. Associations were presented as prevalence ratios (PRs) with 95% CIs. There was no missing data for the variables included in this analysis. All analyses were conducted using Stata 15.0 (StataCorp, College Station, TX).

## Results

The study was conducted between March 2013 and March 2016. During this time, a total of 216 WLHIV were enrolled for care at the Couples Counseling Center. Of these, 172 (79.6%) were screened for participation in the research cohort. The main reasons for couples not being screened were 25 (56.8%) declined, 14 (31.8%) were living away from the study area and could not make study appointments, and 5 (11.4%) were considered to be too sick to enroll. Of the WLHIV screened, 159 (92.4%) were enrolled in the study. Their baseline characteristics are presented in Table 1.

**Table 1:** Baseline characteristic of the study sample (N = 159).

Characteristics	Median (IQR) or n (%)
*Sociodemographic characteristics*	
Age	33.7 (26.9–40.5)
Education > 8 years	111 (69.8)
Currently married	142 (89.3)
Tobacco use in past month	3 (1.9)
Drug use in past month	2 (1.3)
*Reproductive characteristics*	
Pregnant by urine human chorionic gonadotropin test	10 (6.3)
Number of children	2 (1–2)
Fertility desire	99 (62.3)
Fertility intent	40 (25.2)
Uses a modern non-barrier contraceptive method	39 (24.5)
Any condom use in the past week	82 (51.6)
New sex partner(s) in past month	4 (2.5)
Any STIs	5 (3.1)
*Partner attitude towards pregnancy*	
Excited	99 (62.3)
Would not care	33 (20.8)
Very upset	17 (11.3)
*Any controlling behavior by current partner*	66 (41.5)
*Depressive symptoms by patient health questionnaire-9 score >9 (indicating major depressive disorder)*	7 (4.4)
*Alcohol use problems by alcohol use disorder identification test score*	
Non-drinkers (score = 0)	119 (74.8)
Minimal (score = 1–6)	32 (20.1)
Moderate/severe (score >7)	8 (5.0)
*History of violence by someone other than current partner*	
Any physical violence since age 15	23 (14.5)
Any sexual violence since age 15	12 (7.6)
*Any intimate partner violence in the past year*	47 (29.6)
Any physical intimate partner violencein the past year	27 (17.0)
Any emotional intimate partner violence in the past year	32 (20.1)
Any sexual intimate partner violence in the past year	27 (17.0)

Participants’ median age was 33 years (interquartile range [IQR] 29–38), and more than two thirds had attended school for more than eight years (111, 69.8%). The majority (142, 89.3%) were currently married, and few were pregnant (10, 6.3%). Three quarters of the participants (119, 74.6%) did not drink alcohol. Among the 40 participants with any alcohol use, AUDIT scores were consistent with minimal alcohol use in 32 (80.0%) women, while 8 (20.0%) had AUDIT scores suggestive of moderate/severe drinking. While most participants reported that their partners would be excited (99, 62.3%) if they became pregnant, some reported that they would not care (33, 20.8%), and a few reported they would be upset (17, 11.3%). Partner controlling behavior was reported by more than a third of the participants (66, 41.5%).

When asked about violence since the age of 15 years by someone other than the current partner, 23 (14.5%) reported physical violence and 12 (7.6%) reported sexual violence. Intimate partner violence in the past year was reported by 47 (29.6%, 95% CI 22.9–37.2%) participants. Of these, 32 (68.1%) reported emotional violence, 27 (57.4%) reported physical violence, and 27 (57.4%) reported sexual violence. These percentages add up to more than 100%, as participants could report experiencing more than one type of IPV.

In univariate analyses, pregnancy (prevalence ratio [PR] 2.61, 95% CI 1.61–4.23), alcohol use (minimal drinking PR 2.34, 95% CI 1.47–3.73, moderate/severe drinking PR 2.04, 95% CI 0.64–4.29), any male partner controlling behavior (PR 2.48, 95% CI 1.50–4.12), history of physical violence by someone other than the current partner (PR 2.51, 95% CI 1.61–3.90), and history of sexual violence by someone other than the current partner (PR 1.79, 95% CI 0.96–3.34) were associated with a higher prevalence of IPV in the previous year (Table 2).

**Table 2:** Correlates of any IPV in the past 12 months by the current partner.

Variable	Any IPV n (%) or median (IQR) n = 47	No IPV n (%) or median (IQR) n = 112	PR (95% CI)	p-value	Wald p-value	APR (95% CI)	p-value	Wald p-value
Age	32.0 (26.8–37.2)	34.4 (27.2–41.6)	0.97 (0.93, 0.99)	0.05		0.99 (0.95, 1.03)	0.47	
Education > 8 years	32 (68.1)	79 (70.5)	0.92 (0.55, 1.54)	0.76				
Currently married	45 (95.7)	97 (86.6)	2.69 (0.71, 10.12)	0.14				
Tobacco use in past month	2 (4.3)	1 (0.9)	2.31 (1.00, 5.34)	0.50				
Drug use in the past month	2 (4.3)	0	No convergence	NA				
Patient health questionnaire-9 Score >9 (indicating major depressive disorder)	3 (6.4)	4 (3.6)	1.48 (0.61, 3.61)	0.39				
Pregnant by urine human chorionic gonadotropin test	7 (14.9)	3 (2.7)	2.61 (1.60, 4.24)	<0.01		2.14 (1.09, 4.20)	0.03	
Number of children	2.0 (1.0)	2.0 (1.0)	1.07 (0.91, 1.25)	0.43				
Fertility desire	33 (70.2)	66 (58.9)	1.43 (0.84, 2.44)	0.19				
Fertility intent	14 (29.8)	26 (23.2)	1.26 (0.75, 2.10)	0.37				
Contraceptive use	12 (25.5)	27 (24.1)	1.05 (0.61, 1.83)	0.85				
New sex partner(s) in past month	1 (2.0)	3 (3.0)	0.84 (1.51, 4.70)	0.85				
*Partner attitude towards pregnancy*								
Excited (ref)	28 (59.6)	71 (63.4)	1.00					
Would not care	9 (19.1)	24 (21.4)	0.65 (0.20, 2.09)	0.47	0.74			
Very upset	3 (6.4)	14 (12.5)	1.04 (0.55, 1.97)	0.91				
Male partner controlling behavior	30 (63.8)	36 (32.1)	2.48 (1.50, 4.12)	<0.001		2.09 (1.24, 3.51)	0.005	
Physical violence since age 15 by someone else	14 (29.8)	9 (8.0)	2.51 (1.61, 3.91)	<0.001		1.93 (1.22, 3.05)	0.005	
Sexual violence since age 15 by someone else	6 (12.8)	6 (5.4)	1.79 (0.96, 3.35)	0.07		1.30 (0.61, 2.74)	0.50	
*Alcohol use problems by alcohol use disorder identification test score*								
Non-drinkers (ref)								
Minimal	27 (57.4)	92 (82.1)	1.0			1.0		
Moderate/	17 (36.2)	15 (13.4)	2.34 (1.47, 3.73)	<0.001	0.002	1.91 (1.10, 3.33)	0.02	0.06
High	3 (6.4)	5 (4.5)	2.04 (0.64, 4.29)	0.30		1.17 (0.53, 2.59)	0.71	

Each additional year of age (PR 0.97, 95% CI 0.93–0.99) was associated with a lower prevalence of IPV in the past year. In the multivariate model, pregnancy (adjusted prevalence ratio [aPR] 2.14, 95% CI 1.09–4.20), alcohol use (minimal aPR 1.91, 95% CI 1.10–3.33, moderate/severe aPR 1.17, 95% CI 0.53–2.59), male partner controlling behavior (aPR 2.09, 95% CI 1.24–3.51), and history of physical violence by someone other than the current partner (aPR 1.93, 95% CI 1.22–3.05) remained significantly associated with higher prevalence of IPV in the past year.

Past physical and sexual violence by someone other than the current partner were collinear, so the less strongly associated sexual violence variable was not included in the multivariate model. Finally, in the multivariate analysis, the association between age and IPV was substantially attenuated (aPR 0.99, 95% CI 0.95–1.03).

## Discussion

This study examined the prevalence and correlates of IPV among Kenyan women with HIV in serodifferent relationships. Nearly one third of women had experienced IPV in the past year. The prevalence of IPV was substantially higher in women who were pregnant and in those who reported any alcohol use. In addition, controlling behavior by the current partner and history of violence by someone other than the current partner were associated with a higher prevalence of IPV.

While few studies have examined the prevalence of IPV in women with HIV in serodifferent relationships, a number of studies provide estimates of IPV prevalence in African women with HIV enrolled in care. Two studies from Uganda found prevalence of past-year IPV in the range of 30%, which is similar to the prevalence identified in this study of Kenyan women [32]. In contrast, a third study from Uganda found a prevalence of past-year IPV of 7.5% among women with HIV in care [33].

Differences in the prevalence of IPV in different studies need to be interpreted in the context of the specific instruments used to collect data [34]. The current analysis used the WHO questionnaire with behaviorally specific questions on acts of IPV, which has been found to improve disclosure rates [35]. In contrast, the Ugandan study with lower prevalence used the conflict tactics scale, which does not include questions about specific types of physical or sexual IPV and does not include measures of emotional IPV [33].

In this cohort of Kenyan women with HIV in serodifferent relationships, pregnancy was associated with a higher prevalence of IPV in the past year. During pregnancy, women’s sexual behavior may change as a result of reduced sexual desire associated with hormonal changes and difficulty in sexual positioning due to increased abdominal size, leading to marital conflict and IPV [36, 37]. A previous cohort study conducted among serodifferent couples in Africa found a weaker association between pregnancy and IPV [38]. Direct comparison of these studies is difficult, as the cohort study combined both non-pregnant and pregnant follow-up time for women who were pregnant at any time during follow-up.

Alcohol use was associated with a higher prevalence of IPV in this cohort of women with HIV in serodifferent relationships. Even minimal alcohol use, which was by far the largest category other than abstinence, was significantly associated with IPV. Moderate or severe alcohol use was uncommon, and had a smaller point estimate for association with IPV, but with wide confidence intervals. It is interesting that even minimal alcohol use was associated with IPV, a finding with several possible explanations. A bi-directional association between alcohol use and IPV is plausible, as women may use alcohol to cope with IPV [13]. Additionally, women who use alcohol are more likely to have partners who use or abuse alcohol, putting these women at a higher risk for IPV [13]. Further, women who consume alcohol may experience IPV when the male partner perceives their alcohol use as inappropriate and the women may be unable to negotiate non-violent solutions to conflict, or may initiate conflict which results in IPV [13].

The association between male partner controlling behavior and IPV observed in this cohort is consistent with the findings of prior studies [6, 12, 39]. Some authors have argued that men with controlling behavior dominate the relationship, leading to conflict in which male partners exert their power and control in the form of IPV [39].

In the present analysis, a history of physical violence by someone other than the current partner was associated with a higher prevalence of IPV in the past year. This finding is consistent with previous studies showing that women with a history of violence are at elevated risk of to IPV because they are more likely to meet abusive partners, and use alcohol [13, 14]. Another possible explanation is that women exposed to violence as punishment during childhood/adolescence may perceive that this is a normative way of solving conflict, making them more likely to submit to IPV [40].

The high prevalence of IPV in this cohort of women with HIV in serodifferent relationships underscores the need to roll out interventions to address this important public health concern. Several interventions aimed at reducing IPV have been evaluated [41–43]. In general, the most effective approaches have been multifaceted interventions designed to change entrenched gender norms [42, 43].

Strengths of this study included the use of questions adapted from the WHO VAW survey. This is a standardized instrument used successfully in a range of populations of women in Africa, facilitating comparison to other studies [27]. In addition, potential correlates of IPV were measured using validated tools such as the AUDIT score to measure alcohol use [26]. Furthermore, the present analysis focused on IPV in the past year, emphasizing that IPV is a continuing problem for women with HIV in serodifferent relationships.

This study had some limitations. First, we did not include male partners. Therefore, only limited male partner characteristics (e.g. male controlling behavior) were collected from the female participants, and data such as alcohol use by the male partner was not collected. On the other hand, enrolling male partners could have resulted in women being less willing to participant and/or report experiences of IPV if they knew that their male partners were participants. Second, this study design was not able to ascertain temporal and causal relationships between some of the correlates and IPV. For instance, this study did not examine the temporal relationships between pregnancy and IPV. In this context, it is not possible to distinguish between the hypotheses that pregnancy led to IPV versus sexual IPV resulting in pregnancy. Nevertheless, this analysis shows that women with HIV in serodifferent relationships may be at risk for IPV during pregnancy. Third, there may have been underreporting of IPV due to recall bias or social desirability bias. To address this concern, questions specific to behavior were asked to facilitate disclosure [27, 35]. Fourth, this study was conducted in a cohort that was recruited in Nairobi, Kenya, and may not be generalizable to all African WLHIV in serodifferent relationships. The findings are likely to be most generalizable to other WLHIV in serodifferent relationships in urban settings in Kenya and the surrounding region. Finally, it should be noted that the study was not intended to examine whether being in a serodifferent relationship was associated with a higher prevalence of IPV compared to couples where both partners are seronegative or seropositive.

In conclusion, this study identified a high prevalence of IPV in the past year among women with HIV in serodifferent relationships. Women who were pregnant and those who had experienced prior violence had a higher prevalence of IPV. These data highlight the need to screen for IPV during clinic visits, and to offer evidence based interventions for reducing IPV to affected women in serodifferent relationships.

## Supporting information

S1 Checklist STROBE statement—checklist of items that should be included in reports of cross-sectional studies. (DOCX)

S2 Checklist Inclusivity of global research questionnaire. (DOCX)

S1 Data Database used for analysis. (SAV)

## List of abbreviations

aPR: Adjusted prevalence ratio
ART: Antiretroviral treatment
AUDIT: Alcohol Use Disorder Identification Test
CI: Confidence Intervals
HIV: Human Immunodeficiency Virus
IPV: Intimate partner violence
IQR: Interquartile range
KNH: Kenyatta National Hospital
PHQ: Patient Health Questionnaire
PR: Prevalence ratio
STI: Sexually transmitted infections
VAW: Violence against women
VCTs: Voluntary Counseling and Testing Centers
WHO: World Health Organization
