# Supplementary material for: Prevalence and correlates of intimate partner violence among women with HIV in serodifferent relationships in Nairobi, Kenya
Source: PLoS One. 2022 Aug 17;17(8):e0272640. doi: 10.1371/journal.pone.0272640 (PMC9385022; doi:10.1371/journal.pone.0272640)
Supplement: S1 Checklist — (DOCX) [file pone.0272640.s001.docx]

S1 checklist: STROBE Statement—Checklist of items that should be included in reports of *cross-sectional studies*

|  | Item No | Recommendation |
| --- | --- | --- |
| **Title and abstract** | 1 | (*a*) Indicate the study’s design with a commonly used term in the title or the abstract. **Response:** This is included in the abstract, page 3, line 38. |
|  |  | (*b*) Provide in the abstract an informative and balanced summary of what was done and what was found.  **Response:** This has been included in the methods and the results section of the abstract on page 3, lines 38 to 51. |
| Introduction | | |
| Background/rationale | 2 | Explain the scientific background and rationale for the investigation being reported  **Response**: This was included in the introduction section of the manuscript on pages 5 to 7, lines 62 to 110. |
| Objectives | 3 | State specific objectives, including any prespecified hypotheses.  **Response:** The objective of the study was included on page 7, lines 109 to 110 of the manuscript. |
| Methods | | |
| Study design | 4 | Present key elements of study design early in the paper.  **Response:** We reported the study design in the abstract and in the first sentence in the methodology section on page 3, line 38 and page 7, lines 113 to 114 respectively. |
| Setting | 5 | Describe the setting, locations, and relevant dates, including periods of recruitment, exposure, follow-up, and data collection.  **Response:** We have included the study setting and location in the methods section, on page 7, lines 114 to 116. The period of recruitment and data collection has been included in the results section on page 11, line 202. |
| Participants | 6 | (*a*) Give the eligibility criteria, and the sources and methods of selection of participants. **Response:** We have included this information in the recruitment and enrolment procedures on page 7, lines 126 to 133. |
| Variables | 7 | Clearly define all outcomes, exposures, predictors, potential confounders, and effect modifiers. Give diagnostic criteria, if applicable.  **Response:** We have defined all outcomes, exposures, and correlates in the data analysis section on pages 10 to 11 of the manuscript. |
| Data sources/ measurement | 8* | For each variable of interest, give sources of data and details of methods of assessment (measurement). Describe comparability of assessment methods if there is more than one group.  **Response:** We described these in the data analysis section on pages 10 to 11 of the manuscript. |
| Bias | 9 | Describe any efforts to address potential sources of bias.  **Response:** We described potential sources of bias and their mitigations in the limitation section on page 19, lines 316 to 318. |
| Study size | 10 | Explain how the study size was arrived at.  **Response**: “Sample size calculation was not performed specifically for this secondary analysis, which used data from an existing study.” We have included this information on the study design section on page 7, lines 118 to 120 of the manuscript. |
| Quantitative variables | 11 | Explain how quantitative variables were handled in the analyses. If applicable, describe which groupings were chosen and why.  **Response**: We described how the quantitative variables were handled in the data analysis section on pages 10 to 11 of the manuscript. |
| Statistical methods | 12 | (*a*) Describe all statistical methods, including those used to control for confounding. **Response**: We described how the statistical methods were handled in the data analysis section on pages 10 to 11 of the manuscript. |
|  |  | (*b*) Describe any methods used to examine subgroups and interactions. NA |
|  |  | (*c*) Explain how missing data were addressed.  **Response:** “There was no missing data for the variables included in this analysis.” We have included this information in the data analysis section on page 11, lines 198 to 199. |
|  |  | (*d*) If applicable, describe analytical methods taking account of sampling strategy. NA |
|  |  | (*e*) Describe any sensitivity analyses. NA |
| Results | | |
| Participants | 13* | 1. Report numbers of individuals at each stage of study—eg numbers potentially eligible, examined for eligibility, confirmed eligible, included in the study, completing follow-up, and analysed.   **Response:** We have included these information in the results section page 11, lines 203 to 209. |
|  |  | (b) Give reasons for non-participation at each stage.  **Response:** We have included these information in the results section page 11, lines 202 to 207. |
|  |  | (c) Consider use of a flow diagram. NA |
| Descriptive data | 14* | (a) Give characteristics of study participants (eg demographic, clinical, social) and information on exposures and potential confounders.  **Response:** We included these information in the results section on pages 11 to 13, lines 202 to 226. |
|  |  | (b) Indicate number of participants with missing data for each variable of interest. **Response:** “There was no missing data for the variables included in this analysis.” We have included this information in the data analysis section on page 11, lines 198 to 199. |
| Outcome data | 15* | Report numbers of outcome events or summary measures.  **Response:** We included these information in the results section pages 11 to 16. |
| Main results | 16 | (*a*) Give unadjusted estimates and, if applicable, confounder-adjusted estimates and their precision (eg, 95% confidence interval). Make clear which confounders were adjusted for and why they were included.  **Response:** We included these information in the results section pages 13 to 16, lines 227 to 243. |
|  |  | (*b*) Report category boundaries when continuous variables were categorized.  **Response:** We included this information in the data collection section page 9, lines 162 to 167. |
|  |  | (*c*) If relevant, consider translating estimates of relative risk into absolute risk for a meaningful time period. NA |
| Other analyses | 17 | Report other analyses done—eg analyses of subgroups and interactions, and sensitivity analyses. NA |
| Discussion | | |
| Key results | 18 | Summarise key results with reference to study objectives.  **Response:** This information was included in the discussion section on pages 16 to 20. |
| Limitations | 19 | Discuss limitations of the study, taking into account sources of potential bias or imprecision. Discuss both direction and magnitude of any potential bias.  **Response:** This information was included in the discussion section on page 19, lines 307 to 325. |
| Interpretation | 20 | Give a cautious overall interpretation of results considering objectives, limitations, multiplicity of analyses, results from similar studies, and other relevant evidence.  **Response:** We have included these information in the discussion section pages 16 to 20. |
| Generalisability | 21 | Discuss the generalisability (external validity) of the study results.  **Response:** This information has been included in the discussion section on page 19, lines 306 to 324. |
| Other information | | |
| Funding | 22 | Give the source of funding and the role of the funders for the present study and, if applicable, for the original study on which the present article is based.  **Response:** This information was included in the Financial Disclosure section of the submission form as per the journal requirements for publication. |

*Give information separately for exposed and unexposed groups.

**Note:** An Explanation and Elaboration article discusses each checklist item and gives methodological background and published examples of transparent reporting. The STROBE checklist is best used in conjunction with this article (freely available on the Web sites of PLoS Medicine at http://www.plosmedicine.org/, Annals of Internal Medicine at http://www.annals.org/, and Epidemiology at http://www.epidem.com/). Information on the STROBE Initiative is available at www.strobe-statement.org.
